# Vitamin D Status during Pregnancy in a Multi-Ethnic Population-Representative Swedish Cohort

**DOI:** 10.3390/nu8100655

**Published:** 2016-10-22

**Authors:** Linnea Bärebring, Inez Schoenmakers, Anna Glantz, Lena Hulthén, Åse Jagner, Joy Ellis, Mattias Bärebring, Maria Bullarbo, Hanna Augustin

**Affiliations:** 1 Department of Internal Medicine and Clinical Nutrition, Sahlgrenska Academy, University of Gothenburg, 40530 Gothenburg, Sweden; lena.hulthen@medfak.gu.se (L.H.); hanna.augustin@gu.se (H.A.); 2 Medical Research Council Human Nutrition Research, CB19NL Cambridge, UK; I.Schoenmakers@uea.ac.uk; 3 Department of Medicine, Faculty of Medicine and Health Science, University of East Anglia, NR47TJ Norwich, UK; 4 Antenatal Care, Närhälsan Primary Care, 41118 Gothenburg, Sweden; anna.glantz@vgregion.se (A.G.); ase.jagner@vgregion.se (Å.J.); 5 Antenatal Care, Närhälsan Primary Care, Södra Bohuslän, 41119 Gothenburg, Sweden; joy.ellis@vgregion.se; 6 Gothenburg Technical Institute, 41125 Gothenburg, Sweden; mattias.barebring@gti.se; 7 Södra Älvsborgs Hospital, Department of Obstetrics and Gynaecology, 50182 Boras, Sweden; maria.bullarbo@vgregion.se; 8 Department of Obstetrics and Gynaecology, Sahlgrenska Academy, University of Gothenburg, 41684 Gothenburg, Sweden

**Keywords:** pregnancy, vitamin D status, vitamin D deficiency, Sweden

## Abstract

There is currently little information on changes in vitamin D status during pregnancy and its predictors. The aim was to study the determinants of change in vitamin D status during pregnancy and of vitamin D deficiency (<30 nmol/L) in early pregnancy. Blood was drawn in the first (T1) and third trimester (T3). Serum 25-hydroxyvitamin D (25(OH)D) (*N* = 1985) was analysed by liquid chromatography tandem-mass spectrometry. Season-corrected 25(OH)D was calculated by fitting cosine functions to the data. Mean (standard deviation) 25(OH)D was 64.5(24.5) nmol/L at T1 and 74.6(34.4) at T3. Mean age was 31.3(4.9) years, mean body mass index (BMI) was 24.5(4.2) kg/m^2^ and 74% of the women were born in Sweden. Vitamin D deficiency was common among women born in Africa (51%) and Asia (46%) and prevalent in 10% of the whole cohort. Determinants of vitamin D deficiency at T1 were of non-North European origin, and had less sun exposure, lower vitamin D intake and lower age. Season-corrected 25(OH)D increased by 11(23) nmol/L from T1 to T3. The determinants of season-corrected change in 25(OH)D were origin, sun-seeking behaviour, clothing style, dietary vitamin D intake, vitamin D supplementation and recent travel <35° N. In conclusion, season-corrected 25(OH)D concentration increased during pregnancy and depended partly on lifestyle factors. The overall prevalence of vitamin D deficiency was low but common among women born in Africa and Asia. Among them, the determinants of both vitamin D deficiency and change in season-corrected vitamin D status were fewer, indicating a smaller effect of sun exposure.

## 1. Introduction

During the past decade, vitamin D has received increasing attention and has been associated with health benefits in addition to its recognized effects on bone health. One of these areas of interest is vitamin D status during pregnancy. The pregnant woman’s vitamin D status determines the vitamin D status of her newborn infant [1]. Poor maternal vitamin D status during pregnancy is associated with lower bone mineral density and muscle strength in the infant [2]. Poor maternal vitamin D status has also been associated with pregnancy complications such as preeclampsia [3], premature birth [4] and infants born small for gestational age [5]. In Sweden, approximately 3% of pregnancies are complicated by preeclampsia [6] and just below 5% of singletons are born prematurely [7]. We have previously shown that 25-hydroxyvitamin D (25(OH)D) concentrations in the third trimester and changes in 25(OH)D during pregnancy are associated with lower odds of preeclampsia [8].

Studies show conflicting results concerning whether 25(OH)D changes during pregnancy [9,10,11]. Since data on gestational vitamin D status in populations living at latitudes without seasonality in cutaneous vitamin D synthesis are scarce, a season-corrected analysis is a way of investigating changes in 25(OH)D during pregnancy per se. Such analyses of 25(OH)D concentrations during pregnancy show that vitamin D status between early and late pregnancy tracks moderately and that change depends on supplement use, gestational weight gain and physical activity [12]. 

No population-representative data on 25(OH)D concentrations during pregnancy in Sweden exist. In a previous study, we showed that 17% have <30 nmol/L in the third trimester of pregnancy [13]. Data from other studies show that immigrant women in Sweden are at higher risk of vitamin D deficiency than women of Swedish origin [14,15]. These studies were of small sample size and not representative of the general pregnant population. Danish studies have found average concentrations between 57 and 76 nmol/L and that between 3% and 10% of pregnant women have 25(OH)D levels <25 nmol/L [16,17,18]. Findings from England indicate a mean 25(OH)D of 62 nmol/L [19], while Scottish data indicate a lower mean, 40 nmol/L [20]. National data from Belgium report a mean 25(OH)D of 57 nmol/L and that 12% of pregnant women have 25(OH)D concentrations <25 nmol/L [21]. Population-representative data on vitamin D status during pregnancy is needed to evaluate the potential need for new public-health policies and to provide reliable data on potential risk groups for vitamin D deficiency.

Few studies have investigated vitamin D status longitudinally in a large cohort of pregnant women, and knowledge of change in vitamin D status during pregnancy and its determinants are lacking. Therefore, the aim of this study was to assess 25(OH)D concentrations in the first and third trimester of pregnancy in a population-representative Swedish cohort. Determinants of vitamin D deficiency in the first trimester of pregnancy and of changes in vitamin D status during pregnancy were identified. Subgroup analyses were performed in groups identified to be at higher risk of vitamin D deficiency.

## 2. Materials and Methods 

The GraviD study was designed to investigate the association of vitamin D status during pregnancy with preeclampsia and pregnancy-induced hypertension. Women attending antenatal care in Gothenburg, Södra Älvsborg and Södra Bohuslän in south-western Sweden were eligible for inclusion. The only exclusion criterion was gestational age exceeding 16 weeks at inclusion. Pregnant women of all ages were eligible for inclusion. Recruitment took place during two time periods, the fall of 2013 (2 September–8 November) and the spring of 2014 (24 February–13 June). Study information and consent forms for the participants were provided in eight languages and interpreters were present when required, in line with standard practice of care. In total, 2122 women were included in the study but women who miscarried or terminated the pregnancy, and women who were lost to follow up (i.e., women who moved) were excluded and 1985 women are included in these analyses. This study was conducted according to the Declaration of Helsinki and all procedures were approved by the Regional Ethics Committee in Gothenburg (Dnr 897-11, approved 20 December 2011). Written and informed consent was provided by all participants.

### 2.1. Data Collection

Two blood samples were collected from each participant: the first before gestational week 17 (first trimester; T1) and a second one after gestational week 31 (third trimester; T3). At both time-points, participants answered a questionnaire regarding sun exposure (sun-seeking behaviour and clothing style); recent travel <35° N, intake of foods rich in vitamin D (milk and oily fish at T1 with the addition of margarine, yoghurt and sour milk in T3) [22]; supplement use; and background characteristics not included in the medical charts (education level and country of birth). In Sweden, at the time of data collection, margarine and milk with reduced fat content were fortified with vitamin D3 [23]. Medical charts from antenatal care and obstetric units were retrieved after delivery. Data on employment status at T1, tobacco use at T1 and body mass index (BMI) were collected from the medical charts. 

### 2.2. Laboratory Analysis

Venous blood samples were centrifuged for 10 min within two hours of sampling and sent to the central laboratory at Sahlgrenska University Hospital. The blood samples were kept from sunlight in cardboard boxes, and kept refrigerated until and after transport. Serum was extracted, aliquoted and frozen (56% of the samples were processed within 12 h, 59% within 24 h, 95% within 36 h and 98% within 48 h). There were no differences in serum levels of 25(OH)D depending on time until freezing, consistent with previous findings showing stability of 25(OH)D [24]. Serum was stored at −70 °C until analysis of 25(OH)D. A laboratory analysis of 25(OH)D was performed using liquid chromatography tandem-mass spectrometry (LC-MS/MS; Mass spectrometer API 4000, AB Sciex, Framingham, MA, USA) by the central laboratory in Malmö, Sweden, certified by the Vitamin D External Quality Assessment Scheme (DEQAS). The LC-MS/MS method has a measurement range of 6–450 nmol/L for 25(OH)D3 and of 6–225 nmol/L for 25(OH)D2. Values are given as the sum of 25(OH)D3 and 25(OH)D2. The inter-assay coefficient of variation is 6% at 40 nmol/L for both 25(OH)D3 and 25(OH)D2 [25]. Both samples from each woman were analysed at the same time point. 

### 2.3. Statistical Analysis

Risk factors for vitamin D deficiency (25(OH)D <30 nmol/L) [26] were analysed using a multivariable logistic regression analysis. Cosine functions (for T1 and T3, respectively) were fitted to the data, using mean 25(OH)D concentration of each calendar month. The 25(OH)D concentration was adjusted to the predicted yearly mean 25(OH)D of the function, in order to account for seasonality in vitamin D status. This allows for an analysis of gestational changes in 25(OH)D concentration irrespective of season. Cosine functions were also fitted for the subgroup of women born in Africa and Asia. The determinants of season-corrected change in 25(OH)D during pregnancy (T3–T1) were analysed using a multivariable linear regression analysis. 

Variables included in multivariate analyses were selected on the basis of biological plausibility; the potential determinants of vitamin D deficiency at T1 were season, clothing style, recent travel to <35° N, sun-seeking behaviour, vitamin D supplement use (including multivitamin supplements containing vitamin D), vitamin D intake (from oily fish and milk), origin, BMI at T1, tobacco use at T1 and age. Potential determinants for season-corrected change in 25(OH)D from T1 to T3 were the same as above except season and tobacco use (unavailable at T3). Data collected at T3 were used as appropriate (season, recent travel to <35° N, dietary intake of vitamin D and vitamin D supplement use). Dietary intake of vitamin D at T3 also included margarine, yoghurt and sour milk at T3 (not included in the questionnaire at T1). Also, gestational weight gain was included as a potential determinant. Subgroup analyses of the variables relating to vitamin D deficiency and season-corrected 25(OH)D change were performed for women born in Africa and Asia, using the same models. Confounders in all analyses were parity, educational level at T1 and employment at T1. Also, 25(OH)D at T1 and tobacco use at T1 were considered as confounders in the regression analysis of season-corrected change in 25(OH)D.

Continuous variables were BMI (kg/m^2^), gestational weight gain, dietary vitamin D intake and age. Categorical variables were season (December–February, March–May, June–August, September–November), vitamin D supplement use (any dose and frequency: no/yes), origin (defined as country of birth, categorized as North Europe, America, Continental Europe, Africa and Asia), recent travel to a southern latitude (<35° N within six months of blood sampling: no/yes), sun-seeking behaviour (preference for sun, both sun and shade or shade in sunny weather), clothing style when sunny (often, seldom or never exposes more skin than face and hands to the sun in warm weather), parity (0, 1, 2, ≥3 children), education level (primary, secondary, university), employment (unemployed, fulltime, part time, parental leave) and tobacco use (any: no/yes). Data on eye colour was available, but could not be included in the multivariable analysis due to collinearity with origin.

Difference in means between T1 and T3 was tested using an unpaired Student’s *t*-test for data stratified by season. Agreement between T1 and T3 25(OH)D was assessed using correlation. Computer software IBM SPSS Statistics for Windows version 22.0 (IBM Corp., Armonk, NY, USA) was used for all statistical analyses. 

## 3. Results

### 3.1. Participant Characteristics

In total, 2122 women were included in the GraviD-study. Of these, 118 miscarried or terminated the pregnancy, 13 moved and their medical charts could not be retrieved and six T1 samples were unfit for analysis due to diversions from the study protocol. Hence, a 25(OH)D analysis was performed on 1985 samples at T1. At T3, 1836 blood samples were analysed for 25(OH)D and values were available at both time-points for 1829 women.

Characteristics of the cohort are shown in Table 1. Of the whole cohort, 74% were born in Sweden and 75% in North Europe, 7% in Continental Europe, 6% in Africa (3% in Somalia), 10% in Asia (7% in West Asia) and 2% were born in North or South America. Mean standard deviation (SD) age at inclusion varied only slightly between women born in Africa (29.9(5.7)), North (31.4(4.7)) and Continental Europe (30.9(4.5)), Asia (31.5(5.5)) and North or South America (33.1(3.9)). Mean age of the cohort was 31 years at inclusion with a range of 17.9–47.3.

### 3.2. Vitamin D Deficiency

Overall, 10% of the women had 25(OH)D levels <30 nmol/L (Table 1). Concentrations <30 nmol/L and <50 nmol/L, respectively, were found in 51% and 82% of women born in Africa, 46% and 69% of women born in Asia. Among women born in North Europe, 2% had concentrations <30 nmol/L and 13% <50 nmol/L. 

In a multivariable logistic regression analysis, determinants relating to odds of 25(OH)D concentrations <30 nmol/L at T1 were of non-North European origin, sampling in spring, never exposing skin when sunny, no vitamin D supplementation, lower dietary vitamin D intake and lower age (Table 2). Among the subgroup (*N* = 316) of women born in Africa and Asia, determinants of 25(OH)D concentrations <30 nmol/L were not taking vitamin D supplements, never exposing skin when sunny and lower age (Table 2).

### 3.3. Change in Vitamin D Status during Pregnancy

**Figure 1:**
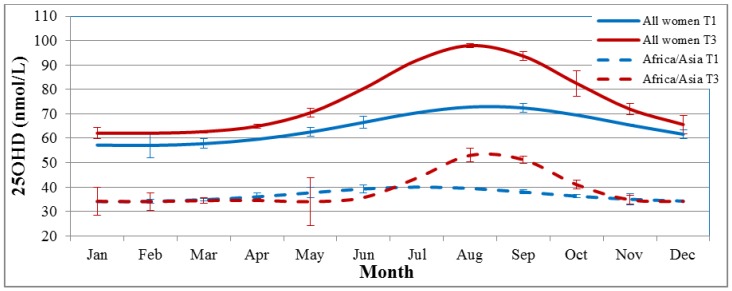
The four cosine functions fitted to the 25(OH)D concentrations at T1 and T3, for the whole cohort and the subgroup born in Africa and Asia. Error bars show the standard deviations of the predicted vs. crude mean 25(OH)D per month. 25(OH)D, 25-hydroxyvitamin D; T1, first trimester; T3, third trimester.

The mean (SD) 25(OH)D concentration was 65 (25) nmol/L at T1 and 75 (34) at T3 and the mean change was an increase of 10 (30) nmol/L. The correlation coefficient between T1 and T3 25(OH)D was *r* = 0.51 (*p* < 0.001). The season-corrected mean 25(OH)D concentration was 64 (24) nmol/L at T1 and 75 (31) nmol/L at T3. The mean season-corrected change during pregnancy was 11 (23) nmol/L. The correlation coefficient between season-corrected 25(OH)D at T1 and T3 was *r* = 0.68 (*p* < 0.001). Season at T3 explained 35% of the variation in uncorrected 25(OH)D change, and 0% of the season-corrected change (adjusted *R*^2^ = 0.35 and 0, respectively). A more asymmetric cosine function for T3 25(OH)D was indicated with a higher amplitude and phase shift than that of the cosine function for T1 (Figure 1).

A season-corrected analysis of change in 25(OH)D showed that the determinants of change in 25(OH)D during pregnancy were origin, sun-seeking behaviour, clothing style, dietary vitamin D intake at T3, vitamin D supplementation at T3 and having travelled to <35° N in the past six months (adjusted *R*^2^ = 0.186) (Table 3). Gestational weight gain, BMI and age were not significantly associated with change in 25(OH)D. In the subgroup of women born in Africa and Asia, only vitamin D supplementation at T3 was a determinant of season-corrected change in vitamin D status during pregnancy (adjusted *R*^2^ = 0.115) (Table 3). 

When performing a cross-sectional analysis based on season, mean 25(OH)D concentrations at T3 were significantly higher than at T1 for all seasons except winter, with the largest difference between T1 and T3 seen during summer (Table 4). 

## 4. Discussion

Overall, our findings show that the prevalence of vitamin D deficiency is low among pregnant women in Sweden. Also, 25(OH)D concentration seems to increase during pregnancy and the impact of season on 25(OH)D concentration seems greater in late pregnancy than in early. The change in season-corrected 25(OH)D during pregnancy is, in part, due to lifestyle factors but most of the variation is yet to be explained. 

Our findings show that the prevalence of vitamin D deficiency is 10% overall and 2% among women born in North Europe. Our data contradict findings from our previous study reporting that 17% of fair-skinned women had concentrations <30 nmol/L in late pregnancy [13]. Vitamin D deficiency among women born in Africa was also less common (50%) than previously indicated (90% among Swedish women born in Somalia) [15]. Risk factors for vitamin D deficiency (25(OH)D <30 nmol/L) in early pregnancy were in line with previous findings [13,17,27]. The determinants in the subgroup born in Africa and Asia were fewer, indicating less effect of season but could possibly also be explained by lower power due to a smaller group size. 

Few studies have previously investigated changes in vitamin D status during pregnancy and results were conflicting. Our results support previous findings from a smaller Swedish study [11] and a study on Gambian women [28] that both show that 25(OH)D concentrations increase during pregnancy. However, Zhang et al. report a downward 25(OH)D trajectory during pregnancy [9]. The only previous study investigating the season-corrected analysis of gestational 25(OH)D change showed decreasing 25(OH)D concentrations [12]. This may partly be explained by less vitamin D supplement use in late pregnancy than in early pregnancy. In our study, the proportion of women using vitamin D supplements was similar at T1 and T3, but it is possible that supplement use contributed to the increase in 25(OH)D if supplementation was initiated shortly before T1. At T1, 43% used a supplement containing vitamin D, including multivitamins and 77% reported taking some supplement (not only vitamin D containing). At T3, 42% used vitamin D containing supplements and 61% in total reported some supplement use. We found that the overall 25(OH)D concentration increased by approximately 10 nmol/L from early pregnancy to the third trimester. This increase was also apparent after 25(OH)D was season-corrected. Similarly, mean 25(OH)D concentrations were higher in the third trimester than in early pregnancy during all seasons except winter, perhaps due to the small group sampled in early pregnancy during winter. We have previously showed that use of estrogen contraceptives is associated with higher 25(OH)D concentrations in non-pregnant women of childbearing age [29]. Similar hormonal changes due to pregnancy cannot be ruled out as a cause of the increasing 25(OH)D concentrations. 

The cosine functions fitted to the data indicate an asymmetrical sinus wave for T3 samples, with a higher seasonal variation in late pregnancy than for T1 samples. This pattern was also seen for the subgroup born in Africa and Asia. Why season would have greater influence on serum concentrations of 25(OH)D in late pregnancy is uncertain, but a similar pattern was also indicated by Moon et al. who performed a similar season-correction of 25(OH)D concentrations [12]. 

We found that determinants of change in season-corrected vitamin D status were related to sun exposure (sun-seeking behaviour, clothing style, and travel <35° N), vitamin D intake (dietary and supplementary) and origin. This model explained about 20% of the variation in season-corrected change in 25(OH)D during pregnancy. Moon et al showed that vitamin D supplementation is related to changes in 25(OH)D between the first and the third trimester, which supports our findings [12]. However, we did not observe the effects of gestational weight gain on 25(OH)D change seen in that study. If this is due to the more heterogeneous study population in terms of ethnicity is unclear. We found that the only determinant of change in the subgroup born in Africa and Asia was vitamin D supplement use. This could be due to less statistical power but could also indicate a smaller effect of sun exposure on 25(OH)D concentrations in this risk group, due to a more concealing clothing style and darker skin pigmentation. 

A limitation of this study is that sampling was overrepresented during autumn and spring, when vitamin D status is highest and lowest, respectively. Balanced sampling during all months of the year might have provided a more precise measure of the seasonality of 25(OH)D concentrations. However, this was, in part, overcome by the season-correction of change in vitamin D status. Another limitation is that ethnicity was defined by country of birth and no information on parental ethnicity was collected. Strengths of this study are the large sample size and the longitudinal study design, alongside the LC-MS/MS method for the 25(OH)D analysis, included in the external quality assurance scheme DEQAS [30]. Almost all pregnant women in Sweden attend the antenatal care, which is free of change. It is therefore an ideal platform for the recruitment of a cohort representative of the pregnant population. The response rate in the study was approximately 33%. In 2012, 24% of women registering for antenatal care were born outside of Sweden, 52% had a university level education, 25% were overweight, 13% were obese, 44% were nulliparous and the mean age was 30 years [7]. The GraviD cohort is thus similar to the population and we thus anticipate that results from this study are likely to be representative of the pregnant population in Sweden. 

## 5. Conclusions 

In conclusion, the overall prevalence of 25(OH)D concentration <30 nmol/L was low in pregnant women in Sweden but common among women born in Africa and Asia. Season-corrected 25(OH)D concentration increased during pregnancy and depends partly on lifestyle factors. Among women born in Africa and Asia, the determinants of both vitamin D deficiency and change in season-corrected vitamin D status were fewer, indicating a smaller effect of sun exposure. These results suggest that targeted public health intervention to treat and prevent vitamin D deficiency in these risk groups is warranted. 

## Figures and Tables

**Table 1:** Characteristics of the study participants.

	Mean	SD
Age (years)	31.3	4.9
Height (cm)	166.8	6.3
Weight T1 (kg)	68.1	12.6
BMI T1 (kg/m^2^)	24.5	4.2
Gestational age T1 (weeks)	10.8	2.0
Gestational age T3 (weeks)	33.4	1.9
25(OH)D T1 (nmol/L)	64.5	24.5
25(OH)D T3(nmol/L)	74.7	34.4
Season-corrected 25(OH)D T1 (nmol/L)	63.6	23.9
Season-corrected 25(OH)D T3 (nmol/L)	74.7	31.3
Vitamin D dietary intake T1 ^2^ (µg/day)	2.4	1.6
Vitamin D dietary intake T3 ^3^ (µg/day)	3.3	1.7
Gestational weight gain ^4^ (kg)	13.5	5.1
	***N*(%)**	
25(OH)D <30 nmol/L, T1	201 (10)	
25(OH)D <50 nmol/L, T1	498 (25)	
Born in Sweden	1479 (74)	
Overweight (BMI 25–29.9) T1 (kg/m^2^)	489 (25)	
Obese (BMI ≥ 30) T1 (kg/m^2^)	203 (10)	
Vitamin D supplement use T1 (any)	868 (43)	
Vitamin D supplement use T3 (any)	842 (42)	
Recently travelled <35° N T1 ^1^	516 (26)	
Recently travelled <35° N T3 ^1^	347 (17)	
Tobacco use at T1 (any)	89 (4)	
University education level T1 (any)	1190 (60)	
Employment T1 (any)	1501 (75)	
Nulliparity T1	836 (42)	

SD, standard deviation; BMI, body mass index; T1, first trimester; T3, third trimester; 25(OH)D, 25-hydroxyvitamin D; ^1^ Travelled to <35° N within six months of the study visit; ^2^ From oily fish and milk at T1; ^3^ From oily fish, milk, margarine, yoghurt and sour milk at T3; ^4^ From gestational week <12 until gestational week ≥35.

**Table 2:** Multivariable logistic regression analysis of the determinants of vitamin D deficiency (<30 nmol/L) in the first trimester (T1) of pregnancy. Data is shown for the whole cohort and for the subgroup born in Africa and Asia.

	All Women ^1^			Women Born in Africa and Asia ^1^		
		95% CI			95% CI	
	OR	Lower	Upper	OR	Lower	Upper
**Origin (continent of birth)**						
North Europe (ref)						
America	5.13 *	1.01	26.15			
Continental Europe	4.55 ***	2.16	9.57			
Asia	22.09 ***	11.51	42.42			
Africa	9.74 ***	4.09	23.18			
**Season T1**						
September–November (ref)						
March–May	2.17 **	1.35	3.49	1.22	0.62	2.41
December–February	1.91	0.29	12.38	10.01	0.63	160.02
June–August	0.59	0.13	2.63	0.22	0.03	1.72
**Vitamin D dietary intake T1**	0.82 **	0.71	0.95	0.84	0.70	1.01
**Sun-seeking behaviour**						
Prefer sun (ref)						
Prefer both sun and shade	0.91	0.48	1.74	0.78	0.33	1.89
Prefer shade	0.64	0.23	1.82	0.47	0.14	1.64
**Clothing when sunny**						
Often expose skin (ref)						
Seldom expose skin	1.68	0.88	3.21	1.44	0.56	3.72
Never expose skin	6.04 ***	2.80	13.02	6.52 ***	2.56	16.60
**Vitamin D supplement T1**						
No (ref)						
Yes	0.09 ***	0.05	0.17	0.04 ***	0.02	0.12
**Travel <35° N before T1**						
No (ref)						
Yes	0.70	0.40	1.22	0.97	0.47	1.98
**BMI T1**	1.02	0.97	1.07	1.01	0.93	1.09
**Age**	0.88 ***	0.83	0.93	0.89 **	0.82	0.96
**Tobacco use T1**						
No (ref)						
Yes	2.18	0.88	5.39	4.71	0.42	52.74

CI, confidence interval; OR, odds ratio; 25(OH)D, 25-hydroxyvitamin D; T1, first trimester; BMI, body mass index; * *p* < 0.05; ** *p* < 0.01; *** *p* < 0.001; ^1^ Adjusted for parity, employment, education level (all at T1).

**Table 3:** Determinants of season corrected change in 25(OH)D during pregnancy (T3–T1), for the whole cohort and the subgroup of women born in Africa and Asia.

	All Women ^1^			Women Born in Africa And Asia ^1^		
	Adjusted *R*^2^ = 0.186			Adjusted *R*^2^ = 0.115		
	Unstandardized Coefficients		P	Unstandardized Coefficients		P
	B	Std. Error		B	Std. Error	
**Origin (continent of birth)**						
North Europe (ref)						
America	−4.61	3.81	0.227			
Continental Europe	−4.75	2.01	0.018			
Africa	−10.95	2.92	<0.001			
Asia	−16.99	2.10	<0.001			
**Sun-seeking behaviour**						
Prefer sun (ref)						
Prefer both sun and shade	−3.18	1.39	0.022	−2.56	3.29	0.437
Prefer shade	−1.13	3.12	0.718	−4.60	4.89	0.348
**Clothing when sunny**						
Often expose skin (ref)						
Seldom expose skin	−4.69	1.57	0.003	−5.85	3.39	0.085
Never expose skin	−7.54	2.80	0.007	−5.37	3.30	0.105
**Vitamin D dietary intake T3 (µg)**	0.99	0.31	0.002	1.07	0.69	0.119
**Vitamin D supplement T3**						
No (ref)						
Yes	16.68	1.08	<0.001	13.67	2.82	<0.001
**Travel <35° N before T3**						
No (ref)						
Yes	3.57	1.31	0.006	−0.92	3.28	0.780
**BMI T1 (kg/m^2^)**	−0.09	0.13	0.489	0.30	0.38	0.301
**Age T1 (years)**	0.22	0.12	0.082	0.27	0.90	0.271
**Gestational weight gain (kg)**	−0.15	0.10	0.126	0.06	0.25	0.805

R^2^, coefficient of determination; B, beta; P, probability; 25(OH)D, 25-hydroxyvitamin D; T1, first trimester; T3, third trimester; BMI, body mass index; ^1^ Adjusted for parity, education level T1 employment status, T1 tobacco use and 25(OH)D at T1.

**Table 4:** Mean and SD 25(OH)D at T1 and T3, and according to season at sampling.

	25(OH)D T1			25(OH)D T3		
Season	*N*	Mean nmol/L	SD	*N*	Mean nmol/L	SD
December–February	43	64	27	344	62	28
March–May	825	58 *	25	633	63 *	28
June–August	77	70 *	21	280	99 *	37
September–November	1040	69 *	24	579	83 *	34
Overall	1985	65	25	1836	75	34

SD, standard deviation; 25(OH)D, 25-hydroxyvitamin D; T1, first trimester; T3, third trimester; * *p* < 0.05 independent samples *t*-test comparing mean 25(OH)D at T1 with mean 25(OH)D at T3, stratified by season.
